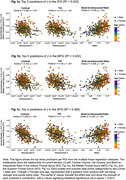# Neuroanatomical predictors of tDCS current distribution in cognitively healthy older adults

**DOI:** 10.1002/alz70856_103172

**Published:** 2025-12-26

**Authors:** Tyler Ann Busch, Kevin Iversen, Skylar Stolte, Gavin Hart, Jason Chen, Pratyush Shukla, Samantha Pedersen, Andrew O'Shea, Yunfeng Dai, Alejandro Albizu, Adam J. Woods, Ruogu Fang, Aprinda Indahlastari

**Affiliations:** ^1^ University of Florida, Gainesville, FL, USA; ^2^ Icahn School of Medicine at Mount Sinai, New York, NY, USA; ^3^ University of South Florida, Tampa, FL, USA; ^4^ University Of Florida, Gainesville, FL, USA; ^5^ The University of Texas at Dallas, Dallas, TX, USA

## Abstract

**Background:**

Transcranial direct current stimulation (tDCS) is a promising intervention for age‐related cognitive decline. Finite element method (FEM) modeling indicates that individual responses to tDCS vary due to individual anatomical differences, affecting delivered current density in the brain. Existing segmentation tools for head tissues are optimized for young adults, inaccurately reflecting older adult anatomy. This study created the largest dataset of manually segmented T1‐weighted images from 367 cognitively healthy older adults to investigate how corrected tissue organization affects tDCS current distribution in the aging brain.

**Method:**

Heads were segmented into 11 tissue types: white matter, gray matter, CSF, air, muscle, fat, skin, blood vessels, eyes, cancellous, and cortical bone. ROAST was modified to accommodate 11 tissues, applying 2mA via electrodes at F4 (anode) and F3 (cathode). Median current densities (J) were calculated for selected regions of interest (ROIs): the Superior Frontal Gyrus (SFG), Inferior Frontal Gyrus (IFG), and Middle Frontal Gyrus (MFG). Multiple linear regression evaluated the relationship between median J, tissue volumes, age, sex, and brain‐to‐intracranial volume across ROIs.

**Result:**

Our regression models explained 49.4% (SFG), 43.2% (IFG), and 43.2% (MFG) of the variance in current density. The most significant predictors were fat, cortical bone, and brain‐to‐intracranial ratio. Fat exhibited a substantial negative relationship with J across ROIs (β range: ‐0.00228 to ‐0.00197, partial η^2^ range: 0.11 to 0.141), as did cortical bone (β range: ‐0.00498 to ‐0.00195, partial η^2^ range: 0.093 to 0.295). Conversely, higher brain‐to‐intracranial volume was positively correlated with Js (β range: 0.00151 to 0.00213, partial η^2^ range: 0.062 to 0.095).

**Conclusion:**

Anatomical variability significantly influences the delivered tDCS current in the brain. Fat had a substantial negative relationship with J across ROIs, likely due to its insulating properties. Similarly, increased cortical volume was associated with decreased J likely due to its resistive properties, causing less current to enter into the intracranial space. Consistent with prior findings, more brain atrophy was associated with less J in the brain, due to current shunting within CSF. These insights underscore the necessity of accurately representing older adult anatomy to personalize tDCS application in older adults.